# Sanhuang Xiexin Tang Ameliorates Type 2 Diabetic Rats via Modulation of the Metabolic Profiles and NF-κB/PI-3K/Akt Signaling Pathways

**DOI:** 10.3389/fphar.2018.00955

**Published:** 2018-08-28

**Authors:** Xiaoyan Wei, Jinhua Tao, Yumeng Shen, Suwei Xiao, Shu Jiang, Erxin Shang, Zhenhua Zhu, Dawei Qian, Jinao Duan

**Affiliations:** ^1^Jiangsu Collaborative Innovation Center of Chinese Medicinal Resources Industrialization, Nanjing University of Chinese Medicine, Nanjing, China; ^2^School of Pharmacy, Nantong University, Nantong, China

**Keywords:** T2DM, Sanhuang Xiexin Tang, NF-κB, PI-3K/AKT, metabonomics

## Abstract

Sanhuang Xiexin Tang (SXT), a classic prescription, has been clinically used to cure diabetes for thousands of years, but its mechanism remains unclear. Here, a systematic in-depth research was performed to unravel how it worked by the signaling pathway and metabonomics analysis. Our studies were conducted using high-fat diets (HFD) and streptozocin (STZ)-induced type 2 diabetes mellitus (T2DM) rats. The blood glucose was measured by a glucose-meter. Protein contents were determined by western blotting or ELISA and mRNA expression was identified by RT-PCR analysis. The pathological status of pancreas was assessed by histopathological analysis. Furthermore, Ultra Performance Liquid Chromatography-Quadrupole-Time of Flight/Mass Spectrometry (UPLC-Q-TOF/MS) coupled with multivariate statistical analysis was performed to discover potential biomarkers and the associated pathways. Hyperglycaemia, insulin resistance, dyslipidemia and inflammation in T2DM rats were significantly ameliorated after 7-week oral administration of SXT. The expressions of phosphatidylinositol-3-kinase (PI-3K), protein kinase B (Akt), glucose transporters-4 (GLUT4) Mrna, and p-PI-3K, p-Akt, GLUT4 protein involved in the PI-3K/Akt signaling pathway of T2DM were markedly up-regulated. Further investigation indicated that the perturbance of metabolic profiling in T2DM rats was obviously reversed by SXT and 38 potential biomarkers were screened and identified. Our study might help clarify the mechanism of SXT and provide some evidences for its clinical application in the future.

## Introduction

Owing to rapidly increasing morbidity and mortality, type 2 diabetes mellitus has become a tremendous threat to human health and one of the most challenges in current medicine. With the growing perceptive of the pathophysiology of T2DM, plentifully pharmacological interventions have been applied to therapy hyperglycemia and interrupt the progression of the disease (Bailey, 2015). However, most of the drugs (acarbose, metformin, gliclazide, rosiglitazone, etc) have certain undesirable side effects such as lactic acidosis, hypoglycemia, edema, etc. (Cheng and Fantus, 2005). Thus, it is necessary to seek effective and novel drugs for preventing or treating diabetes.

Recently, traditional Chinese medicine (TCM) applied to cure diabetes is attracting considerable interests due to its good curative effects, lower toxicity, fewer side effects and lower cost (Li et al., 2004). Many traditional herbal formulas such as *Gegen Qinlian* Tang, *Sarcopoterium spinosum* extract, and *Wuwei Xiaoke* decoction have been clinically used to treat diabetes and worked well (Pang et al., 2015). SXT, a traditional medicinal formula composed of three herbal medicines (*Rhei rhizome*, RR; *Scutellaria radix*, SR; *Coptidis rhizome*, CR) at a ratio of 2:1:1, has been used to cure diabetes since the Tang Dynasty (sixth century C.E.) (Sun, 1999). However, few studies were focused on exploring the possible molecular mechanism of SXT with anti-diabetic activities. TCMs usually accomplish an overall therapeutic effect and reduce drug-related side effects by targeting multiple pathways. Then, a systematic in-depth study using some holistic techniques is needed to confirm the anti-diabetes effects of SXT for the ill-defined mechanisms of this herb medicine. As a platform for comprehensively analyzing the metabolites in entire organism, metabolomic profiling could assess all metabolites in biological samples and provide insights into the holistic efficacy of TCMs (Wang et al., 2013). Among various analytical platforms used for metabonomic analysis, UPLC/Q-TOF-MS is steadily increasing due to its better reproducibility and detection limits, and increased chromatographic resolution (Lenz and Wilson, 2007).

In this study, rats fed with high-fat diet coupled with low-dose STZ-induction, which has been widely used in many experiments to investigate the *in vivo* anti-diabetic effects of various medicines (Skovsø et al., 2015), were selected as the T2DM model. And metformin, currently considered the first-line agent for the pharmacological management of patients with T2DM (Harper et al., 2013), was chosen as the positive control drug. Furthermore, an UPLC-Q-TOF-MS-based metabolomics strategy was used to explore possibly protective effects of SXT on T2DM rats. The present work was very helpful to clarify the pharmacological mechanism of SXT on T2DM.

## Materials and methods

### Preparation of SXT

*Rhei rhizome* (Gansu Province), *Scutellariae radix* (Shanxi Province), and *Coptidis rhizome* (Sichuan Province) were authenticated by Professor Shu Jiang (Nanjing University of Chinese Medicine). The preparation of SXT extract was performed as previously described (Wei et al., 2017). Briefly, the mixture of RR, SR, and CR, blended in a 2:1:1 ratio was extracted three times using water and finally concentrated to 1.0 g/mL.

**Figure 1:**
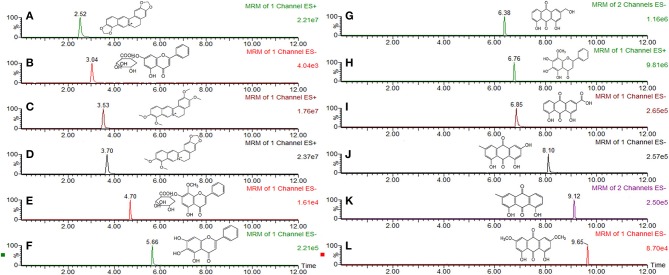
MRM chromatogram and chemical structure of main compounds in Sanhuang Xiexin Tang. **(A)** coptisine, **(B)** baicalin, **(C)** palmatine, **(D)** berberine **(E)** wogonoside, **(F)** baicalein, **(G)** aloeemodin, **(H)** wogonin, **(I)** rhein, **(J)** emodin, **(K)** chrysophanol, **(L)** physcion.

According to the 2015 Edition of Chinese Pharmacopoeia, the main components of SXT included rhein, emodin, chrysophanol, aloeemodin, physcion, baicalin, wogonoside, baicalein, wogonin, berberine, epiberberine, palmatine, and coptisine. Before the experiment, main chemical components of SXT were previously identified by the same UPLC-TQ-MS/MS conditions reported in our earlier publication (Wei et al., 2017). The contents of rhein, emodin, chrysophanol, aloeemodin, physcion, baicalin, wogonoside, baicalein, wogonin, berberine, epiberberine, palmatine, and coptisine in STX were 364.09, 305.11, 460.03, 292.69, 238.72, 612.16, 557.05, 261.51, 74.08, 122.53, 178.41, 109.83 μg·mL^−1^, respectively. Structures and MRM chromatograms of these components were exhibited in Figure 1.

### Animals groups and treatment

Sprague-Dawley male rats (*n* = 63; 180–220 g) bought from Experimental Animal Center of Zhejiang Province (SCXK 20140001) were housed at 21 ± 2°C and 50 ± 10% relative humidity with a 12-h light/12-h dark cycle. All rats were randomly divided into normal (*n* = 7) and model (*n* = 56) groups and provided *ad libitum* access to standard rodent chow and HFD, respectively. The T2DM model was established by feeding HFD for 4 weeks, and then intraperitoneally injected with 35 mg/kg of STZ (Sigma, USA) in 0.1 M citrate buffer (pH 4.2–4.4) (Srinivasan et al., 2005). Seven days after STZ injection, rats with blood glucose values of 16.7–20 mmol/L were considered to be type 2 diabetic rats. All animal experiments were performed following the Guide for the Care and Use of Laboratory Animals, and the procedures were approved by the Research Ethical Committee of Nanjing University of Chinese Medicine.

Rats were assigned to six groups (7 rats/group): normal group (N), T2DM group (M), T2DM rats gavaged with metformin (Met, 200 mg/kg), 5 g/kg SXT (SL), 10 g/kg (SM), and 15 g/kg (SH), respectively. After 7 weeks of treatment, all rats were fasted for overnight and then housed in metabolic cages to collect urine samples. Subsequently, rats were anesthetized with pentobarbital, and then blood samples were collected using heparinised tubes and centrifuged at 3000 rpm for 10 min for obtaining serum. The hepatic tissue and skeletal muscle were rapidly removed, individually washed with isotonic saline and blotted by ash-free filter paper. All samples were stored at −80°C. Pancreases were fixed with 10% formalin for histology estimation.

### Biochemical measurements

Test kits supplied by Nanjing Jiancheng Biological Engineering Institute were used to determine serum levels of triaglycerol (TG), total cholesterol (TC), high-density lipoprotein cholesterol (HDL-C), and low-density lipoprotein cholesterol (LDL-C). Serum concentrations of fast insulin (FINS), C-reaction protein (CRP), tumor necrosis factor-α (TNF-α), interleukin-6 (IL-6), interleukin-1β (IL-1β), and levels of toll-like receptor 4 (TLR4), nuclear factor-kappa-B (NF-κB) in liver tissue were measured by corresponding ELISA kits (Nanjing Jiancheng Bio-Engineering Institute). Insulin sensitivity index (ISI) and insulin resistance index (IRI) were calculated as follows: ISI = –Ln (fasting blood glucose (FBG) × FINS), IRI = FINS × FBG/22.5 (Man et al., 2016).

### Histopathological analysis

Pancreas of six group rats were preserved in 4% paraformaldehyde for 24 h at 4°C, embedded in paraffin and then mounted on silane-covered slides. Subsequently, sectioned pancreatic samples were stained after dewaxing by H and E and analyzed tissue histology via a light microscope (400 × ).

### Real-time PCR

Total RNA was isolated from liver tissues (for analysis of NF-κB, IL-1β, IL-6, TNF-α, and CRP) or skeletal muscle (for analysis of GLUT4, Akt, and PI-3K) by TRIzol reagent (Sigma, St Louis, MO, USA) following the supplier's instructions. cDNA was synthesized by Prime Script™ RT reagent Kit with gDNA Eraser (Takara) according to the manufacturer's instructions. Analysis of qPCR was performed with SYBR Green Master mix with Rox reference dye on an Applied Bio 7500 RT-PCR system. The ΔΔCt value method was used to quantify the relative transcript levels and final results were exhibited as the ratio of mRNA of normal. All genomic sequences of PCR primers were summarized in electronic Supplementary Table 1.

### Western blotting analysis

Skeletal muscle (for analysis of GLUT4, p-Akt, and p-PI-3K) or liver tissues [for analysis of Phospho-IkB Kinase β (p-IKKβ)] were pulverized in lysis buffer [50 mmol/L Tris-HCl (pH 7.5), 150 mmol/L NaCl, 0.5% Sodium deoxycholate, 1% NP-40, 0.1% SDS, 100 μg/mL PMSF, 1 μg/mL Aprotinin (NanJing SunShine Biotechnology Co., LTD.)] for 60 min at 4°C, and then centrifuged at 12,000 rpm for 5 min at 4°C. The proteins were extracted by a total protein extraction kit (Keygen, China) following instructions supplied by the manufacturer and Protein concentrations were ensured by Bio-Rad DC Protein Assay Kit (DC protein assay, BIO-RAD, USA). An equal amount of protein (40 μg) was separated using 10% SDS-PAGE, transferred onto a polyvinylidene difluoride membrane, and then blocked with 0.05% Tween 20 and 5% BSA for 60 min at RT. The immunoblots were incubated with primary antibodies anti-GLUT4 (1:1,000, #2213, CST), anti-phospho-PI-3K (1:1,000, #4228, CST), anti-PI-3K (1:1,000, #4257, CST), anti-phospho-Akt (1:1,000, #4058, CST), anti-Akt (1:1,000, #9272, CST), anti-phospho-IKKβ (1:1,000, ab59195, ABCAM), anti-IKKβ (1:1,000, ab32135, ABCAM), and anti-GAPDH (1:1,000, #2118, CST) in PBS with 1% BSA for overnight, followed by an incubation with secondary antibody (HRP in 1% BSA in PBS, 0.1% Tween 20). Membrane were scanned using LiCor Odyssey Infrared imaging system (LiCor Biosciences, Lincoln, NE, USA) and the signals were detected by a Super ECL plus Kit (Keygen) and protein band intensity was quantified using Image Studio Lite software from LiCor Biosciences.

### Metabolomic study

#### Preparation of sample

Before analysis, samples were thawed at 25 ± 2°C and 600 μL acetonitrile was added to 200 μL aliquot of serum (urine: 600 μL). The mixture was vortexed for 60 s and centrifuged at 13,000 g for 10 min at 4°C to precipitate protein. Then, 600 μL clear supernatants of serum (urine: 1,000 μL) were evaporated to dryness at 37°Cby a centrifugal vacuum concentrator. The residues were reconstituted in 100 μL acetonitrile solution, ultrasonically treated for 60 s and centrifuged as above to obtained clear supernatant for analyzing. The quality control (QC) samples were prepared by pooling and mixing same volume of randomly selected samples from each group, respectively. The QC sample was injected six times before the beginning of analytical run to “condition” or “equilibrate” the system and then every 7 samples to further assess repeatability and stability of the method.

#### UPLC-Q-TOF/MS condition

The analysis was carried out on an ACQUITY™ UPLC BEH C_18_ column (2.1 × 100 mm, 1.7 μm) by Waters ACQUITY UPLC system coupled with Synapt^TM^ Q-TOFMS system (Waters Corporation, USA) which equipped with electrospray ionization (ESI) source. The mobile phase, at a flow rate of 0.4 mL/min, was consisted of 0.1% formic acid-water (A) and acetonitrile (B). Gradient program for serum analysis: 0–3 min, 5% B; 3–11 min, 5–95% B; 11–12 min, 95% B; 12.0 min, 95–5% B. Gradient program for urine analysis: 0–1 min, 5% B; 1–7 min, 5–40% B; 7–10 min, 40–95% B; 10–12 min, 95% B; 12 min, 95–5% B. For both positive and negative electro-spray modes, the condition was selected as follows: capillary voltage, 3.0 kV; extraction cone, 2.0 V; sampling cone, 30 V; source temperature, 120°C and desolvation temperature, 350°C. Nitrogen was used as desolvation and cone gas with the flow rate of 600 and 50 L/h, respectively. ESI^+^ and ESI^−^ of Leucine-enkephalin at *m/z* 556.2771 and *m/z* 554.2615 were used as lock mass for exact mass measurement correction.

#### Data processing and multivariate analysis

Analyzing of raw data was performed by the MarkerLynx software Version 4.1 (Waters, Milford, USA). For data collection, parameters were set as follows: retention time range 0–12 min, mass range 100–1,000 amu, mass tolerance 0.01 Da, retention time tolerance 0.01 min, and noise elimination level 20. Each ion was normalized to the summed total ion intensity per chromatogram, and then resultant data matrices were exported to the EZinfo 2.0 software for obtaining principal component analysis (PCA), supervised orthogonal partial least squares discriminant analysis (OPLS-DA) and PLS-DA. PCA, which could reflect the intrinsic variation in datasets, was used for detecting possible outliers. The quality of OPLS-DA models was described by R^2^Y and Q^2^, which represented the fraction of explained Y-variation and the predictive ability of the model, respectively. Fine models were established when the values R^2^Y and Q^2^ values were above 0.8. The PLS-DA model was used to distinguish the differences among examined groups and identify biomarkers.

S-plot was constructed for visualizing the relationship between covariance and correlation. Metabolites with vital contributions to discrimination between groups were regarded as potential biomarkers. The variable importance in the projection (VIP), which indicated contributions of variables to the discrimination between normal and T2DM rats on the OPLS-DA score plot, was also used to select biomarkers. An independent *t*-test was used to compare the significance difference of these variables between N and M group. The metabolites with VIP >1 and *P* < 0.05 were considered to be potential biomarkers.

#### Biomarker identification and construction of metabolic pathway

The identification of potential biomarkers was according to the *m/z*, retention time, and typical MS/MS fragment of the metabolites, which were gained in positive and negative ion modes. The metabolic pathway interpretation of potential biomarker was performed with Metabo-Analyst 3.0 (http://www.metaboanalyst.ca), a web-based tool for visualization of metabolomics based on database source including the KEGG (http://www.genome.jp/kegg/) and the HMDB (http://www.hmdb.ca/) databases for searching.

### Statistical analysis

Comparison of data was evaluated by student's *t* test. Statistical analyses were assessed by Prism (Version 7.0, GraphPad Software, San Diego, CA, USA). *P* values < 0.05 or 0.01 were considered significant difference.

## Results

### Effects of SXT on hyperglycaemia, insulin resistance and lipid disorder in T2DM rats

**Figure 2:**
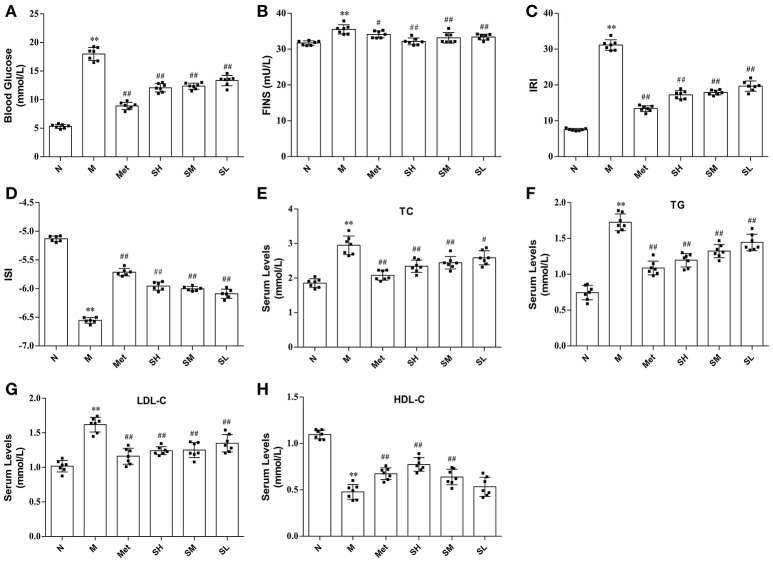
The levels of biochemical indicators in the normal group (N), model group (M), and groups gavaged with metformin (Met), 5 g/kg SXT (SL), 10 g/kg SXT (SM), and 15 g/kg SXT (SH). **(A)** Blood Glucose, **(B)** insulin (FINS), **(C)** insulin resistance index (IRI), **(D)** insulin sensitivity index (ISI), **(E)** total cholesterol (TC), **(F)** triglyceride (TG), **(G)** low-density lipoprotein cholesterol (LDL-C), **(H)** high-density lipoprotein cholesterol (HDL-C). The values were shown as mean ± SD for seven animals. **P < 0.01, *P < 0.05 vs. Normal; ^##^*P* < 0.01, ^#^*P* < 0.05 vs. Model.

The levels of blood glucose, FINS, IRI, ISI, TG, LDL-C, and TC in T2DM model rats were considerably higher than that in normal, which indicated that hyperglycaemia, insulin resistance and lipid metabolism disorders appeared in T2DM rats. While blood glucose, TG, TC, LDL-C levels were significantly decreased, HDL-C concentration was notably increased, and insulin resistance was improved in diabetes rats after oral administration of SXT for 7 weeks (Figure 2).

### Histological effects of SXT on pancreas

**Figure 3:**
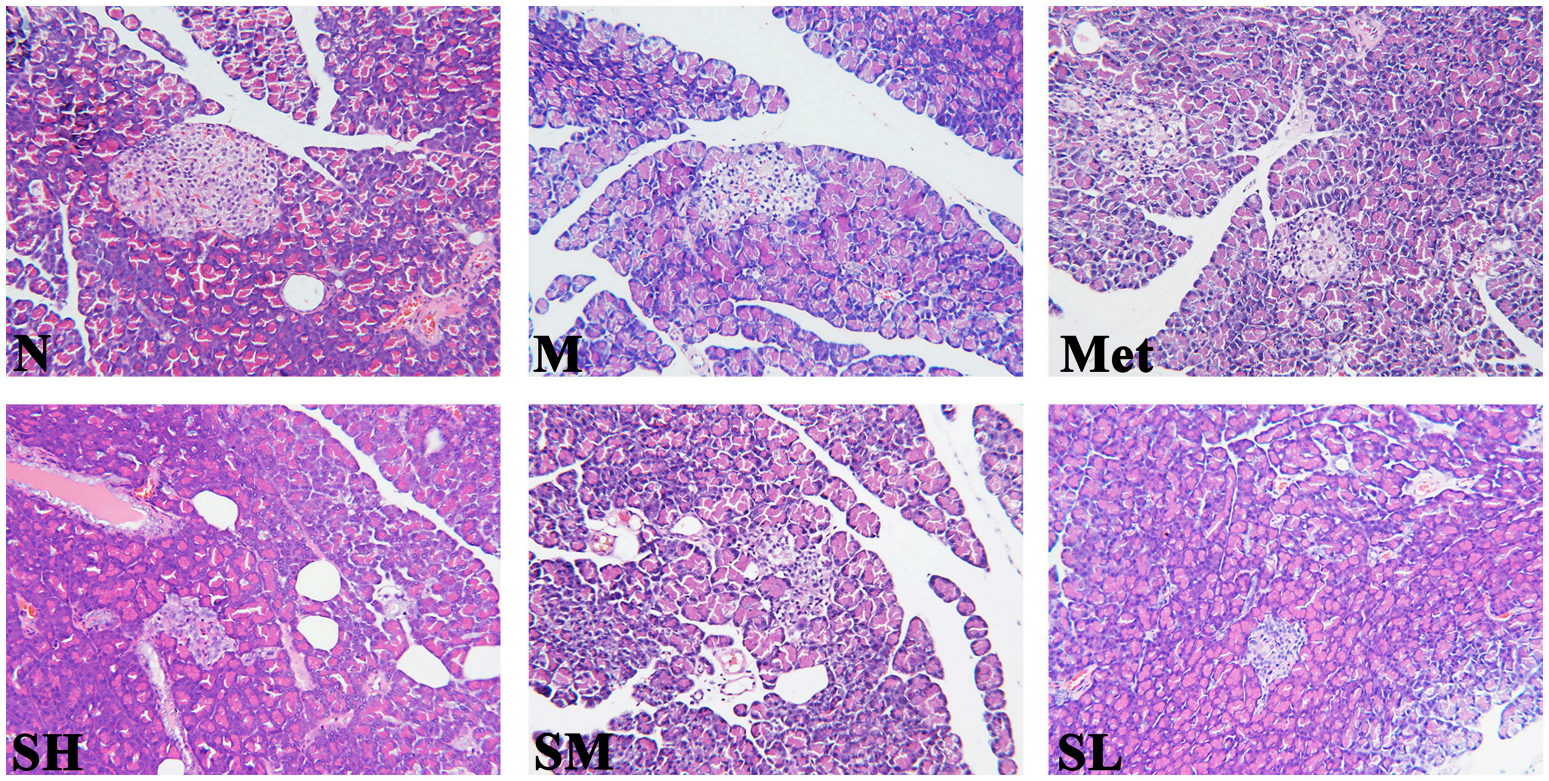
Histopathological observation of pancreas tissues in the normal group (N), model group (M), and groups gavaged with metformin (Met), 5 g/kg SXT (SL), 10 g/kg SXT (SM), and 15 g/kg SXT (SH). Samples were stained with H and E and photographed at 400 × magnification.

Histological analysis was the most direct method for evaluating tissue injury and SXT treatment effects. As compared with normal rats, the islet morphology of T2DM rats was significantly irregular, and pronouncedly endogenous destruction, such as cell degeneration and necrosis, partial ductal vacuolation, islet atrophy, structure of islet in pancreas was unclear, pancreatic β cell numbers reduction and the boundary of islet cells and exocrine gland was blurry, while the abnormal islet structure was obviously ameliorated by SXT in a dose-dependent manner (Figure 3).

### SXT reduced the TLR4, p-ikkβ, nf-κb, and inflammatory cytokines levels in T2DM rats

**Figure 4:**
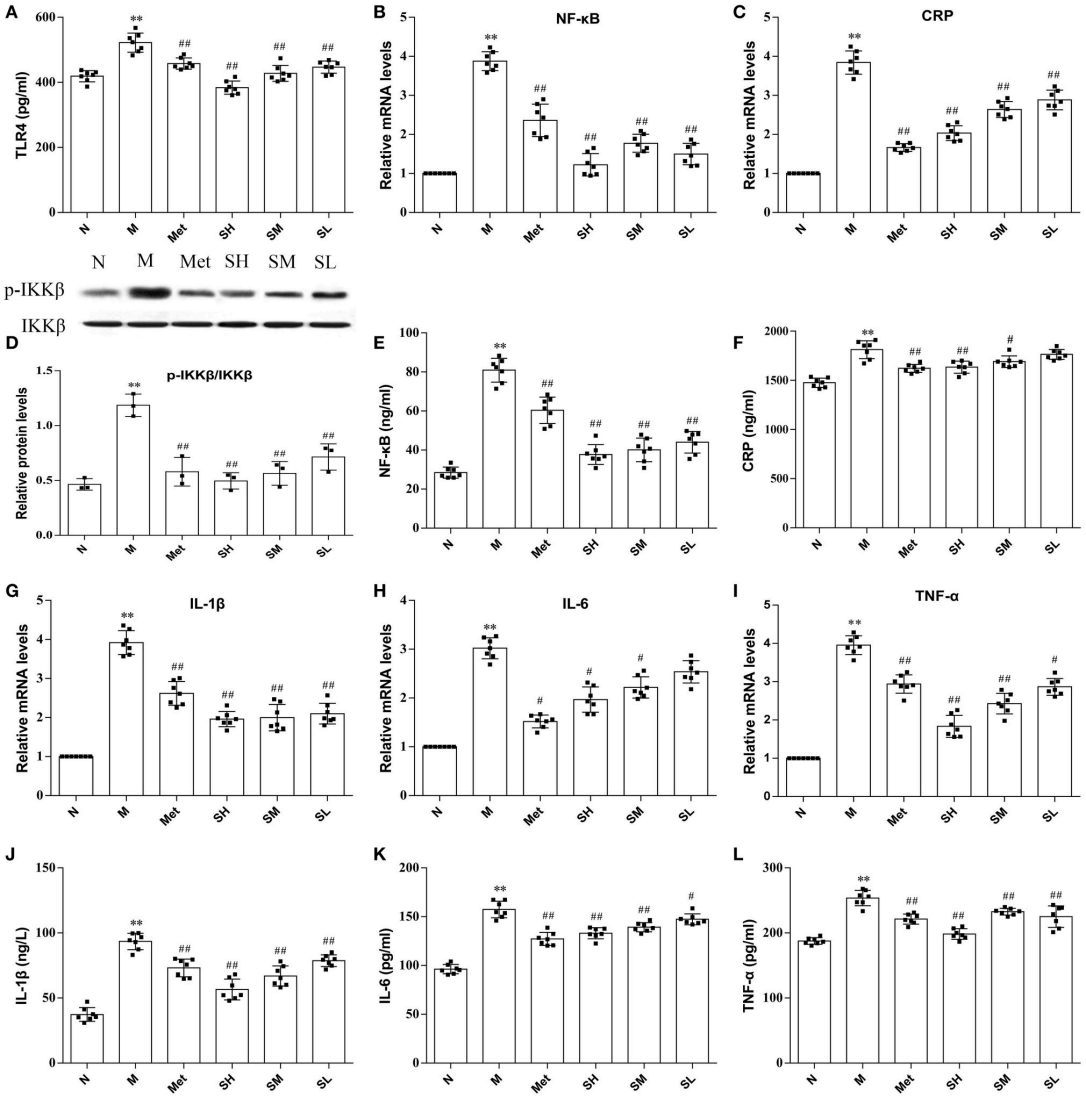
**(A)** Protein level of TLR4 in liver, **(B)** mRNA expression level of nuclear factor kappa-B (NF-κB) in liver, **(C)** mRNA expression level of C-reaction protein (CRP) in liver, **(D)** protein level of p-IKKβ in liver, **(E)** protein level of NF-kB in liver, **(F)** serum content of CRP, **(G)** mRNA expression level of interleukin-1β (IL-1β) in liver, **(H)** mRNA expression level of interleukin-6 (IL-6) in liver, **(I)** mRNA expression level of tumor necrosis factor-α (TNF-α) in liver, **(J)** serum content of IL-1α, **(K)** serum content of IL-6, **(L)** serum content of TNF-α of the normal group (N), model group (M), and groups gavaged with metformin (Met), 5 g/kg SXT (SL), 10 g/kg SXT (SM), and 15 g/kg SXT (SH). The values were shown as mean ± SD for seven animals. **P < 0.01, *P < 0.05 vs. Normal; ^##^*P* < 0.01, ^#^*P* < 0.05 vs. Model.

TLR4, a key immune receptor, plays a vital role in stimulating inflammation. Dose-dependent effects of SXT on suppressing the increased expression of TLR4 in T2DM rats were exhibited in Figure 4A. Given that CRP, IL-6, IL-1β, and TNF-α were widely recognized as markers of inflammation, serum levels of these cytokines were measured. The results illustrated that higher levels of inflammatory cytokines were found in model rats than normal rats and the contents of IL-6, IL-1β, CRP, and TNF-α in T2DM rats notably decreased after oral administration of SXT (Figures 4F,J–L). To further support our observation that the activation of inflammatory cytokines in T2DM rats could be effectively inhibited by SXT, mRNA levels of CRP, IL-6, IL-1β, and TNF-α in rat liver were identified by RT-PCR. Results exhibited that higher mRNA levels of CRP, IL-6, IL-1β, and TNF-α in diabetes rats were obviously reduced after the treatment with STX for 7 weeks (Figures 4C,G–I,). Finally, to explore whether the NF-kB pathway mediated SXT-induced inhibition of the inflammatory response, we assessed the activation of p-IKKβ and NF-kB. The extent of IKKβ phosphorylation and concentration of NF-kB were obviously enriched in T2DM rats, but showed significantly lower in SXT-treated groups (Figures 4D,B,E). All these results indicated that the activation of NF-kB pathways in diabetic rats was inhibited by SXT, which might be helpful to decrease inflammation and further improve insulin resistance in T2DM rats.

### SXT activated Pi-3K-mediated signaling cascade in T2DM rats

**Figure 5:**
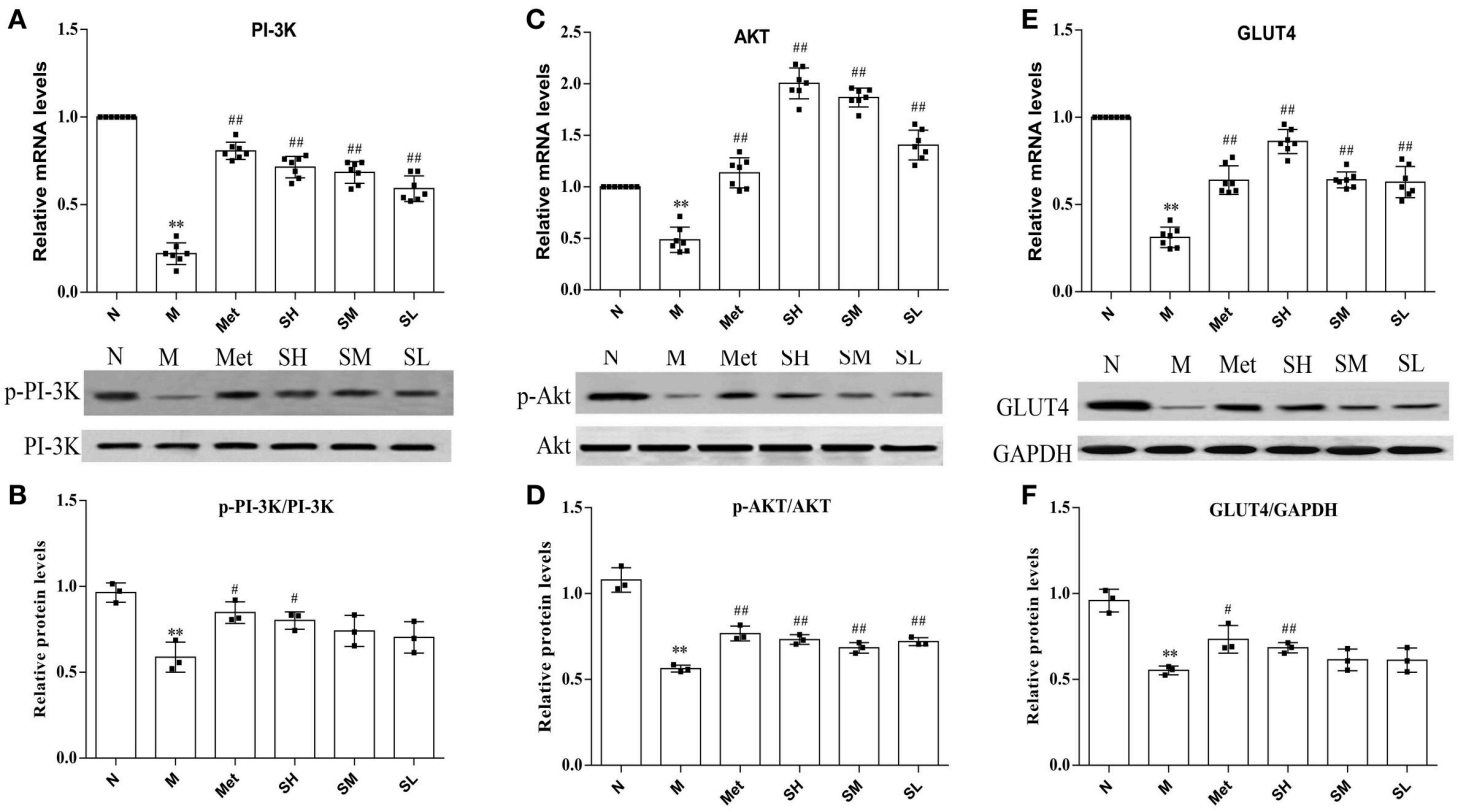
**(A)** mRNA expression level of PI-3K, **(B)** mRNA expression level of Akt, **(C)** mRNA expression level of GLUT4, **(D)** protein expression level of phosphorylated PI-3K, **(E)** protein expression level of phosphorylated Akt and **(F)** protein expression level of GLUT4 in skeletal muscle of the normal group (N), model group (M), and groups gavaged with metformin (Met), 5 g/kg SXT (SL), 10 g/kg SXT (SM), and 15 g/kg SXT (SH). The values were shown as mean ± SD for seven animals. **P < 0.01, *P < 0.05 vs. Normal; ^##^*P* < 0.01, ^#^*P* < 0.05 vs. Model.

Insulin-stimulated glucose transport is the primary result of GLUT4 translocation, and the PI-3K-mediated pathway plays important roles in insulin stimulated translocation of GLUT4 from intracellular compartment to membrane. To understand the SXT intervention mechanism of glucose uptake in T2DM rats and make clear whether it was associated with PI-3K-mediated pathway, we detected the mRNA levels of GLUT4, PI-3K, Akt, and protein levels of GLUT4, PI-3K, p-PI-3K, Akt, and p-Akt. Results suggested that GLUT4, PI-3K, Akt mRNA levels and protein expression levels of GLUT4, p-PI-3K, p-Akt were obviously inhibited in model group rats than that in normal rats. Diabetes rats, treated with SXT for 7 weeks, showed an upward trend in the expression of GLUT4, phosphorylated Akt and phosphorylated PI-3K in skeletal muscle with a dose-dependence manner (Figures 5A–F). It is illustrated that SXT could enhance key factors in the activation of PI-3K/Akt pathway.

### Metabolic profiling analysis

#### QC samples analysis

**Figure 6:**
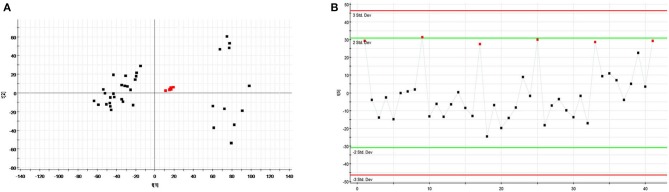
Assessment of QC samples **(A)** PCA score plot of test samples and QC samples; **(B)** Trend plot showing the variation of t_[5]_ over all observations. QC samples were colored as red boxes and test samples were colored as black triangle. X axis numbers represented sample number (41 injections). Y axis was arbitrary (3 s.d.).

The relatively clustering of QCs (Figure 6A) and relative standard deviations (RSD%) of ion intensity (Table 1) expound and prove the quality of QC data, and trend plots showed the variation of t_[5]_ over all observations (Figure 6B). The validation of method was used by Chromatographic peaks of ten ions, which was randomly selected. The examination of method repeatability was used six replicates of QC sample. Our results exhibited that this method had excellent repeatability and stability.

**Table 1:** Coefficient of variation of ion intensity of selected ions present in the QC samples covering the range of retention times.

**t_R_/min**	***m/z***	**QC1**	**QC2**	**QC3**	**QC4**	**QC5**	**QC6**	**RSD%**
4.39	130.0573	56.5378	58.3629	58.1603	57.7151	59.0679	62.8349	3.67
6.09	297.0975	88.5787	91.6349	91.1278	89.7099	86.3001	97.5877	4.21
5.61	170.0598	44.5789	43.3307	45.2503	44.2478	44.3457	46.7744	2.61
9.45	219.1748	25.1735	25.5398	25.5799	25.4988	24.7218	25.1918	1.29
1.03	229.1561	121.4306	124.3145	120.193	123.2513	117.2136	113.4153	3.38
10.36	274.2730	68.6143	65.7671	64.5479	65.9154	66.5988	67.0768	2.07
4.34	302.3177	9.6369	10.1616	9.7275	10.1336	9.8602	10.0973	2.27
3.00	162.0587	25.6528	26.0563	26.5049	25.8306	25.9380	26.1811	1.14
3.61	164.0695	74.6306	67.4420	75.1082	72.7909	70.5433	69.5560	4.21
9.53	149.0436	95.0513	98.4203	96.6133	95.0106	102.2311	97.0951	2.77

#### Data analysis

Typical total ion chromatograms of serum and urine samples, collected from N, M, SL, SM, and SH group rats in positive and negative ESI, were showed in Supplementary Figure 1. However, the difference of five group rats in same biological specimen was not clear.

**Figure 7:**
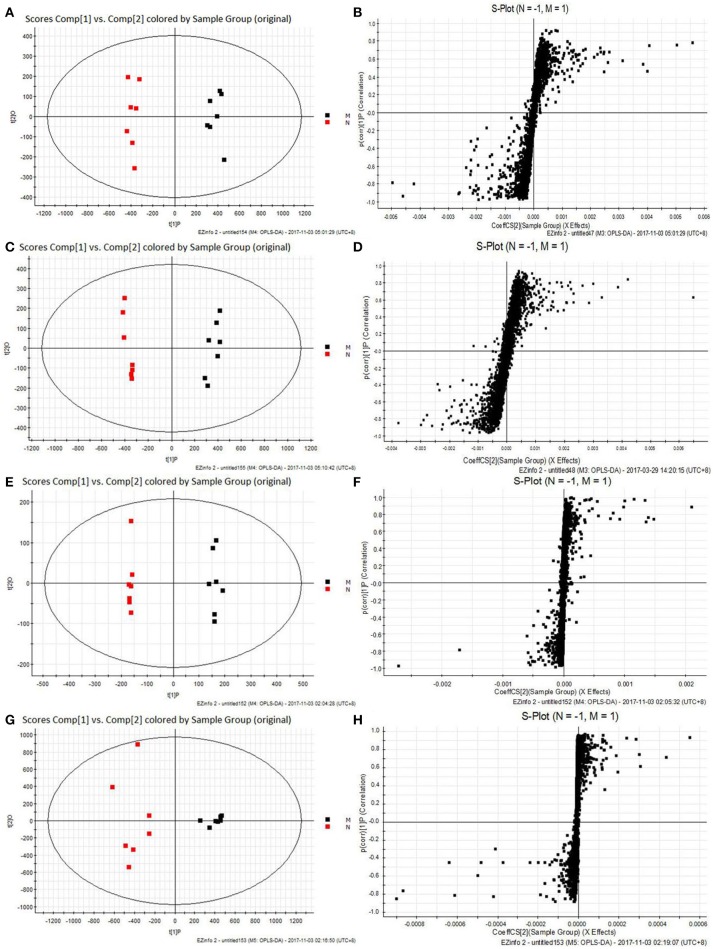
The results of PCA score plots and S-plots of OPLS-DA models. **(A,B)** Serum in ESI^+^ mode; **(C,D)** Serum in ESI^−^ mode; **(E,F)** Urine in ESI^+^ mode; **(G,H)** Urine in ESI^−^ mode. normal group (N), and model group (M) (A, R^2^Y = 0.9884, Q^2^ = 0.8880; C, R^2^Y = 0.9816, Q^2^ = 0.0.8901; E, R^2^Y = 0.9971, Q^2^ = 0.9261; G, R^2^Y = 0.9937, Q^2^ = 0.9727).

All the raw data were imported in Masslynx™ software for multiple statistical analyses to obtain a comprehensive view of the metabonome. The gained PCA scores plots exhibited a clear clustering of samples obtained from the N and M group (Figures 7A,C,E,G), which demonstrated that N and M groups were obviously separated. The R^2^Y and Q^2^ of OPLS-DA model in ESI^+^ and ESI^−^ modes for serum and urine samples were suggested that the OPLS-DA model was good to fitness and prediction, and then the OPLS-DA score plots (Figures 7B,D,F,H) were used to find the potential metabolites associated with T2DM progress. Subsequently, combining the results of the S-plot and VIP-value together, the UPLC-Q-TOF/MS platform supplied the retention time, molecular mass and MS/MS data for further structural identification of biomarkers.

#### Changes of relative intensity of endogenous metabolites

**Figure 8:**
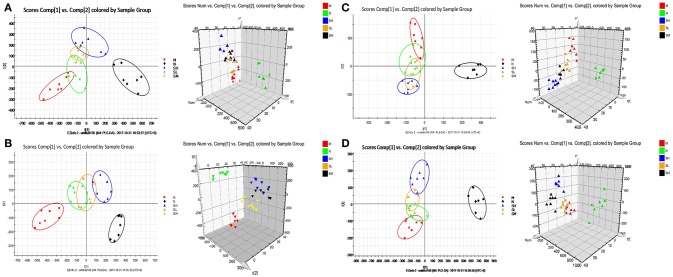
PCA scores plot resulting from UPLC/Q-TOF-MS spectra. **(A)** Serum samples at positive ion mode. **(B)** Serum samples at negative ion mode. **(C)** Urine samples at positive ion mode. **(D)** Urine samples at negative ion mode. normal group (N), model group (M), and groups gavaged with metformin (Met),5 g/kg SXT (SL), 10 g/kg SXT (SM), and 15 g/kg SXT (SH).

**Figure 9:**
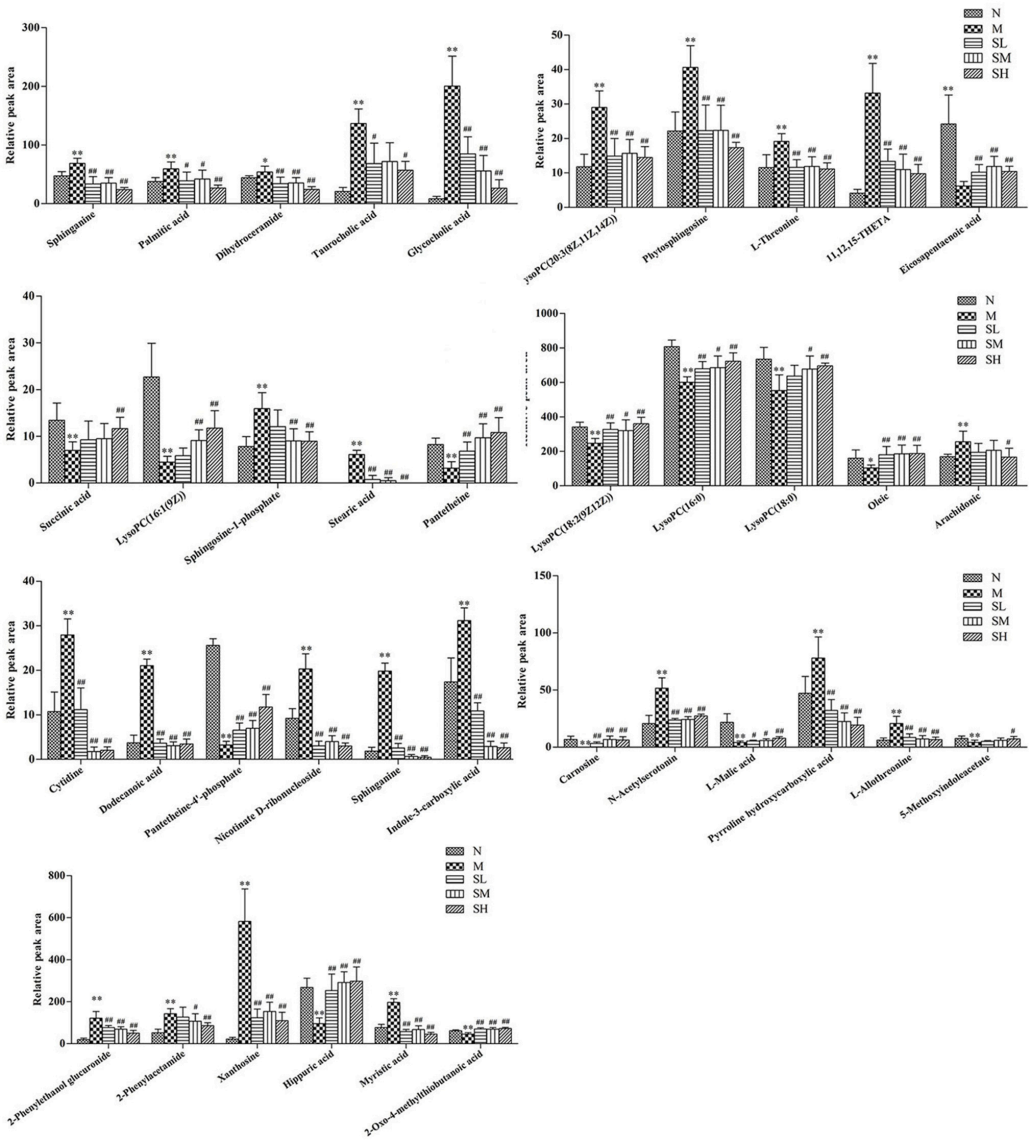
Effects of Sanhuang Xiexin Tang on characteristic biomarkers. normal group (N), model group (M), and groups gavaged with metformin (Met), 5 g/kg SXT (SL), 10 g/kg SXT (SM), and 15 g/kg SXT (SH).**P < 0.01, *P < 0.05 *vs*. Normal; ^##^*P* < 0.01, ^#^*P* < 0.05 *vs*. Model.

To investigate the effects of SXT on T2DM rats, a PLS-DA method was applied to evaluate the changes among N, M, SH, SM, and SL groups. The results indicated that M group were obviously separated from N group, and three SXT-treated groups showed the obvious separation from M group and were closer to N group than M group in the direction of principal component (Figure 8), which suggested that SXT altered the metabolism of T2DM rats in certain degree. Furthermore, among the endogenous metabolites, which were finally identified by comparing with the authentic standards or according to the protocol detailed above method, 38 biomarkers (twenty in serum and eighteen in urine) were highly reversed after treatment of SXT for 7 weeks. The detailed information was showed in Tables 2, 3 and Figure 9.

**Table 2:** Potential serumal biomarkers of SXT treated type 2 diabetes rats.

**t_R_ min**	***m/z***	**HMDB ID**	**VIP**	**Metabolite**	**Trend^a^**	**KEGG pathway**
5.43	119.1516	00254	1.06	Succinic acid	↓	Alanine, aspartate and glutamate metabolism, Propanoate metabolism, Butanoate metabolism, TCA cycle
5.93	302.3067	00269	2.61	Sphinganine	↑	Sphingolipid metabolism
6.43	494.3283	10383	2.13	LysoPC [16:1 (9Z)]	↓	Glycerophospholipids
7.55	546.3601	10394	1.97	LysoPC [20:3 (8Z,11Z,14Z)]	↑	Glycerophospholipids
6.97	520.3411	10386	1.64	LysoPC [18:2 (9Z,12Z)]	↓	Glycerophospholipids
4.56	274.2756	00220	1.47	Palmitic acid	↑	Biosynthesis of unsaturated fatty acids, Fatty acid biosynthesis, Fatty acid elongation in mitochondria, Fatty acid metabolism
4.63	318.3029	04610	2.10	Phytosphingosine	↑	Sphingolipid metabolism
7.62	496.3199	10382	4.96	LysoPC (16:0)	↓	Glycerophospholipids
9.41	524.3340	10384	2.02	LysoPC (18:0)	↓	Glycerophospholipids
1.24	120.0895	00167	1.21	L-Threonine	↑	Glycine, serine and threonine metabolism, Aminoacyl-tRNA biosynthesis
7.46	330.3412	06752	2.64	Dihydroceramide	↑	Sphingolipid metabolism
7.56	313.2802	04684	1.32	11,12,15-THETA	↑	Arachidonic acid metabolism
9.78	301.2201	01999	2.50	Eicosapentaenoic acid	↓	Fatty acids biosynthesis
5.89	378.2416	00277	2.44	Sphingosine 1-phosphate	↑	Sphingolipid metabolism
8.94	283.2432	00827	2.25	Stearic acid	↑	Fatty acid biosynthesis
3.28	514.2856	00036	3.08	Taurocholic acid	↑	Bile secretion, Fatty acid biosynthesis, glycolysis and gluconeogenesis, metabolism of lipids
3.69	464.3061	00138	2.21	Glycocholic acid	↑	
11.20	281.2466	00207	7.03	Oleic acid	↓	Biosynthesis of unsaturated fatty acids, Fatty acid biosynthesis
10.77	303.2296	01043	6.29	Arachidonic acid	↑	Arachidonic acid metabolism
9.94	277.1985	03426	1.62	Pantetheine	↓	Pantothenate and CoA biosynthesis

*Trend^a^ Models vs. Controls: ↑ indicated increase; ↓ indicated decrease*.

**Table 3:** Potential urinary biomarkers of SXT treated type 2 diabetes rats.

**t_R_ min**	***m/z***	**HMDB ID**	**VIP**	**Metabolite**	**Trend^a^**	**KEGG pathway**
4.34	302.3177	00269	1.78	Sphinganine	↑	Sphingolipid metabolism
3.00	162.0587	03320	4.07	Indole-3-carboxylic acid	↑	Amino acids metabolism
9.61	227.1862	00033	1.13	Carnosine	↓	Histidine metabolism
9.45	219.1748	01238	3.14	N-Acetylserotonin	↑	Tryptophan metabolism
1.03	229.1561	00806	2.08	Myristic acid	↑	Fatty acid biosynthesis
2.26	135.0627	00156	1.62	L-Malic acid	↓	Pyruvate metabolism,TCA cycle
4.39	130.0573	01369	1.38	Pyrroline hydroxycarboxylic acid	↑	Arginine and proline metabolism
1.41	120.0829	04041	1.43	L-Allothreonine	↑	Glycine, serine and threonine metabolism
2.04	206.0295	04096	2.02	5-Methoxyindoleacetate	↓	Tryptophan metabolism
9.53	149.0436	01553	1.61	2-Oxo-4-methylthiobutanoic acid	↓	Cysteine and methionine metabolism
6.09	297.0975	10350	6.81	2-Phenylethanol glucuronide	↑	Pentose and glucuronate interconversions, Starch and sucrose metabolism
3.36	242.0066	00089	6.75	Cytidine	↑	Pyrimidine metabolism
7.51	199.1030	00638	2.52	Dodecanoic acid	↑	Fatty acid biosynthesis
3.47	134.0604	10715	5.78	2-Phenylacetamide	↑	Phenylalanine metabolism
4.35	283.0675	00299	13.67	Xanthosine	↑	Purine metabolism
7.60	357.0754	01416	3.56	Pantetheine 4′-phosphate	↓	Pantothenate and CoA biosynthesis
3.51	255.0845	06809	3.47	Nicotinate D-ribonucleoside	↑	Nicotinate and nicotinamide metabolism
3.00	178.0462	00714	8.01	Hippuric acid	↓	Phenylalanine metabolism

*Trend^a^ Models vs. Controls: ↑ indicated increase; ↓ indicated decrease*.

#### Metabolic pathway analysis

**Figure 10:**
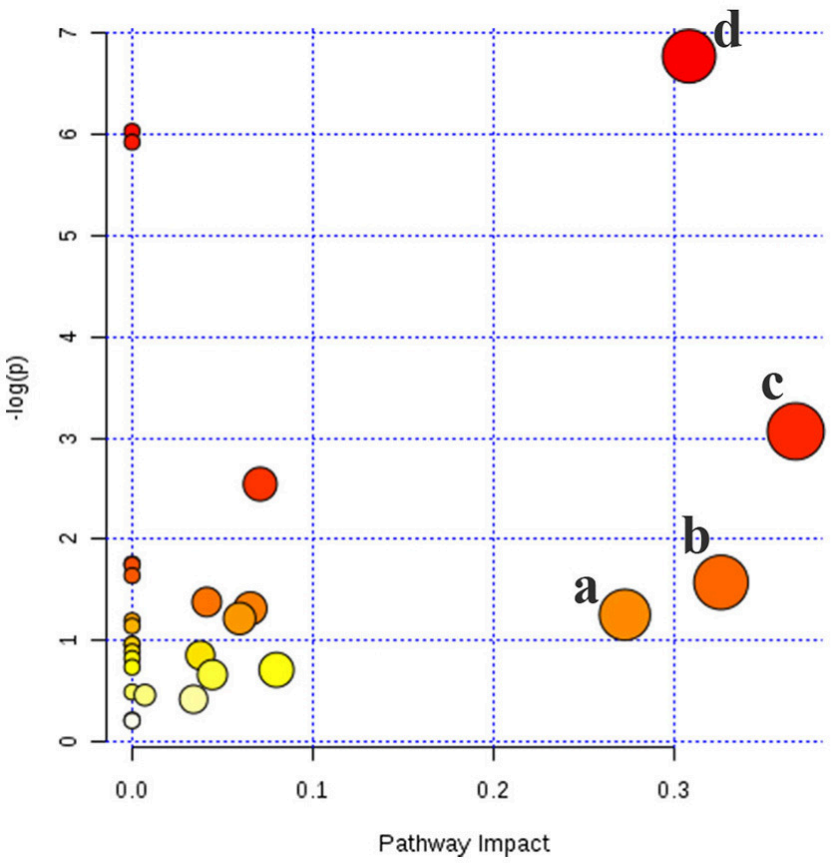
Metabolic pathways involved potential markers in serum and urine. **(a)** Pentose and glucuronate interconversions, **(b)** Arachidonic acid metabolism, **(c)** Pantothenate and CoA biosynthesis, **(d)** Sphingolipid metabolism.

**Figure 11:**
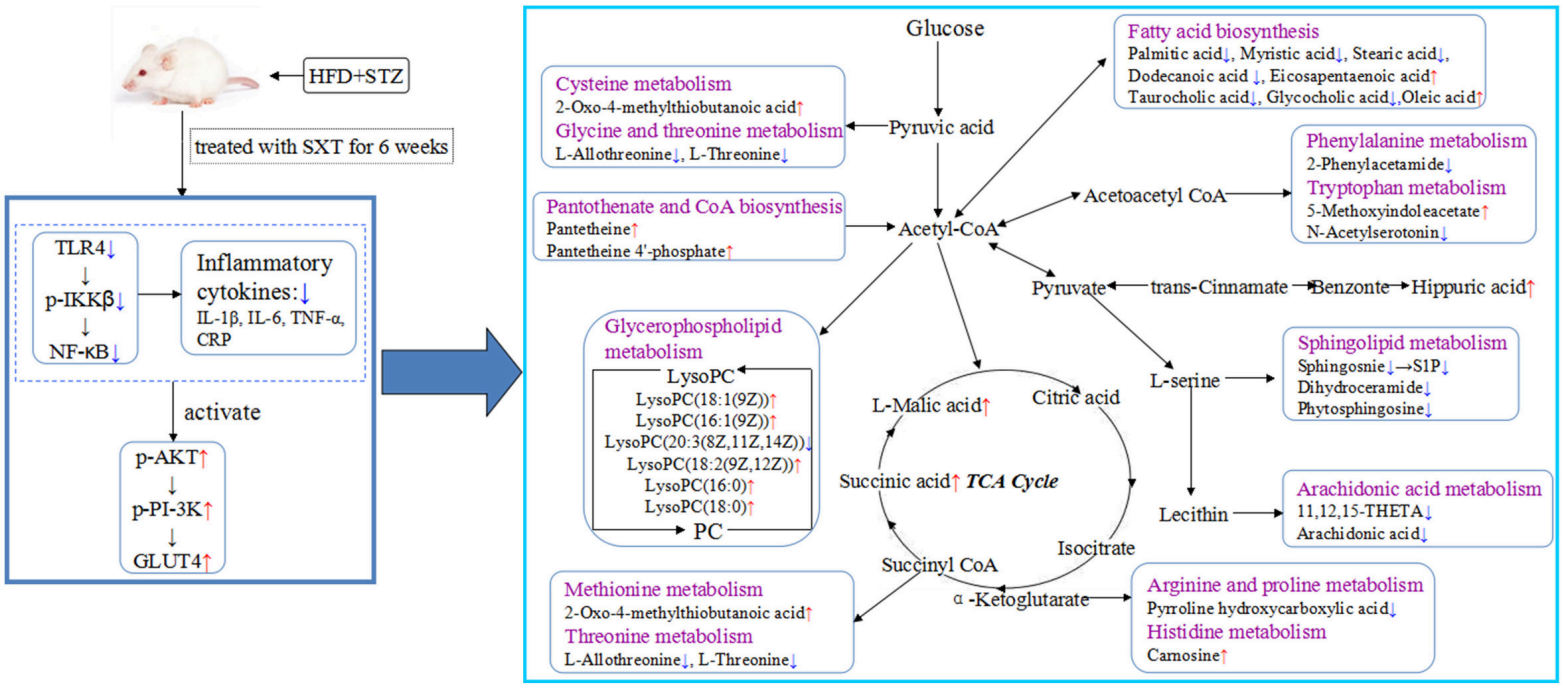
Systemic view of metabolic pathways associated with SXT treatment in T2DM rats. (Compared with the model group, the red arrows represented the increase of the contents of endogenous metabolites, while the blue arrows represented the decrease of the contents of endogenous metabolites. Purple colors indicated the names of the related metabolic pathways).

Based on Metabo Analyst 3.0, the pathway impact value was calculated from pathway to topology analysis. As shown in Figure 10, pentose and glucuronate interconversions, arachidonic acid metabolism, pantothenate, and CoA biosynthesis and sphingolipid metabolism were considered as the most important metabolic pathways. Additionally, for revealing the correlation among distinct potential biomarkers, associated pathways of these metabolites were explored by the KEGG and HMDB databases. A correlation network of potential metabolites related to effects of treatment on T2DM was exhibited in Figure 11.

## Discussion

T2DM is a common chronic metabolic disease resulting from insulin resistance, impaired insulin secretion or both (Zimmet et al., 2001). Accumulating studies indicate that chronic inflammation is considered an important driver of insulin resistance and type 2 diabetics (Hotamisligil, 2006), and IKK-NF-κB is one of the principal inflammatory pathways that disrupt insulin action (Cai et al., 2005). A variety of researches have showed that plasma LPS levels seem to rise with higher fat intake in mice (Cani et al., 2007). The binding of LPS with TLR4 could result in the stimulation of IKKβ and NF-κB and then increase the levels of downstream inflammatory factors (like TNF-α, IL-6, IL-1β, etc.) (Kagan and Medzhitov, 2006; Wan et al., 2016), which promoted the insulin resistance in the tissue where they were produced. Our data suggested that SXT could suppress the activation of IKKβ/NF-κB signal pathway in liver and decrease the CRP, TNF-α, IL-6, and IL-1β levels in serum of T2DM rats, which were corresponding with the results that insulin resistance was markedly ameliorated in SXT-treated diabetes rats. As reported, liver has an architectural organization, in which hepatocytes has immediate access to a vast network of blood vessels and then establish the communications with other tissues such as pancreatic islets and muscle (Hotamisligil, 2006). Therefore, the amelioration of inflammation and insulin resistance by SXT in liver of T2DM rats might contribute to activate insulin signaling cascades in skeletal muscle, which accounted for more than 80% of insulin induced glucose disposal in humans (Zierath et al., 1996). Then, we subsequently investigated the influences of SXT on transduction pathways of the insulin effects.

The translocation and redistribution of GLUT4 from an intra-cellular compartment to the plasma membrane, which needs activation of PI-3K and Aktthat mediated by insulin receptor (Welsh et al., 2005; Ueda-Wakagi et al., 2015), is the critical step of insulin-stimulated glucose uptake(Petersen and Shulman, 2006). Then, levels of GLUT4, phosphorylated PI-3K, and phosphorylated Akt are major indexes that reflect the activity of PI-3K/Akt pathway, which plays a vital role in insulin-activated glucose uptake in muscle tissue and inhibiting glucose release from hepatocytes (Khan and Pessin, 2002). Results in this study illustrated that the decreased abundance of p-PI3K, p-Akt, and GLUT4 in T2DM rats were highly up-regulated by SXT, which might indicate that SXT could promote the uptake and utilization of glucose by activating the PI-3K/Akt/GLTU4 pathway and subsequently reduce the blood glucose level in T2DM rats. Based on the above analysis, SXT could effectively ameliorate glycaemic and insulin resistance which were both key focuses in the management of T2DM (Holman et al., 2008).

As diabetes was a metabolic disease, we investigated the effects of SXT on metabolic disorders in T2DM rats to depict more insights into mechanism. Identified as a quantitative determination of the dynamic multi-parametric metabolic reaction of biological systems to pathophysiological stimulation or genetic modification (Qi et al., 2014b), metabonomics has been considered as an effectively tool for investigating the mechanisms of TCMs (Wang et al., 2012; Qi et al., 2014a). Moreover, it is well known that serum metabolites could reflect the systemic physiological changes and metabolites of urine are terminal products of biological metabolism. Therefore, the plasma and urine metabolomics research, which can discover potential biomarkers and the correlated pathways of disease, will be useful for clarifying the mechanism of SXT in improving metabolic disturbance of T2DM rats.

In our study, five glycerophospholipids {LysoPC[20:3 (8Z,11Z,14Z)], LysoPC[16:1(9Z)], LysoPC[18:2(9Z,12Z)], LysoPC(16:0), and LysoPC(18:0)}, showing abnormal changes in T2DM rats, were highly intervened by SXT treatment. Glycerophospholipids, the essential constituents in lipid bilayer of cells, have a vital function in cell communication on the signaling processes and metabolism. Numerous researches have suggested that LysoPCs, which could activate the adipocyte glucose intake and increase the glucose levels of diabetes mice (Yea et al., 2009), play a vital role in glycolipid metabolism and the development of some metabolic diseases such as inflammation, diabetes, cancer, and hyperlipemia (Jackson et al., 2008; Kabarowski, 2009). Additionally, Low LysoPCs were usually regarded as biomarker of insulin resistance (Barber et al., 2012). Thus, the modification of SXT on LysoPCs levels might contribute to improving insulin resistance and dyslipidemia in T2DM rats.

Arachidonic acid (AA) is an indispensable polyunsaturated fatty acid. Studies have illustrated that the esterification of membrane phospholipids in sn-2 position, which was activated by physicochemical or pathological factors, could generate AA (Funk, 2001). As a precursor of many compounds with potently pro-inflammatory and anti-inflammatory functions, AA plays a key role in inflammation (Buczynski et al., 2009). Furthermore, AA has also been reported to have the effects in promoting or inhibiting the production and secretion of insulin in islet-cells (Wörmann et al., 2012). Normal levels of AA could promote the synthesizing and secreting of insulin. But, super-physiological levels of AA could lead to the apoptosis of islet β-cells and then reduce the synthesizing and secreting of insulin (Jiang et al., 2017). Therefore, the promotion of AA level might contribute to increase the risk of diabetes. In this study, data illustrated that the concentration of AA was obviously higher in T2DM rats than normal rats. The down-regulated effects of SXT on the increased AA level in diabetic rats might contribute to protecting islet β-cells, modifying insulin release and alleviating inflammation.

As reported, hydrolyzation of sphingomyelin by sphingomyelinases would lead to the releasing of ceramide and accumulating of phytosphingosine (Pavoine and Pecker, 2009). Sphingosine-1-phosphate (S1P), a metabolic product of ceramide, plays a major role in stimulating the inflammatory pathways (Spiegel and Milstien, 2002). Moreover, the elevated level of S1P in serum illustrated that the metabolism of sphingolipid increased, which might decrease reverse cholesterol transport pathway and then enhance the risk of hyperlipidemia (Liu et al., 2014). Elevation of phytosphingosine with critical roles in sphingolipids biosynthesis and metabolism were observed in the T2DM rat serum, which suggested that the expression of sphingomyelinases and metabolism of sphingolipid were both increased in diabetes rats. After intake of SXT, the elevated levels of sphinganine, S1P and phytosphingosine were significantly down-regulated, indicating that SXT could inhibit dysfunctional sphingolipid metabolism to reduce hyperlipidemia and inflammation in T2DM rats.

As the data shown, taurocholic acid and glycocholic acid exhibited the obvious increase in T2DM rats, which corresponded to the prior research that the level of hepatic bile acids was elevated in STZ-induced diabetes (Dong et al., 2009). Taurocholic acid and glycocholic acid were derived from cholic acid, which could influence the fatty acid biosynthesis, glycolysis and gluconeogenesis, metabolism of lipids and lipoproteins (Zhu et al., 2016). The obvious decrease of taurocholic acid and glycocholic acid contents in T2DM rats after oral administration of SXT might be conducive to attenuating lipid disorders and hyperglycaemia.

Succinic acid and L-malic acid, mainly localized in the mitochondria, are significant intermediates of the tricarboxylic acid cycle (TCA), which is acritical pathway for glucose disposal and energy supplying of aerobionts (Zhao et al., 2010). Additionally, concentrations of some endogenous biomarkers, which participated in amino acids metabolism, CoA biosynthesis, fatty acid biosynthesis, were significantly influenced by SXT. As a cofactor for enzymes and participated in 100 different reactions, CoA is necessary in all organisms (Wadler and Cronan, 2007; Snyder et al., 2015). The CoA is a limiting factor for many metabolic processes like TCA cycle, ketogenesis, lipogenesis, and mitochondrial fatty acid import and β-oxidation (Eaton, 2002; Cotter et al., 2013). Hippuric acid, a metabolite of phenylalanine, could be activated by intestinal flora. As reported, concentrations of hippuric acid were lower in HFD-induced hyperlipidemic rats than that in normal (Wu et al., 2014). Many amino acids, served as substrates for protein synthesis, are usually synthesized using TCA intermediates as precursors like α-ketoglutarate for glutamate and amino acids derived by glutamate, and oxaloacetate for amino acids derived by aspartate (Denkert et al., 2008). Recent evidences indicated that blood levels of amino acids were related with the development of type 2 diabetes mellitus (Xu et al., 2013). As the results showed, biomarkers that participated in the metabolism of some essential amino acids were markedly regulated after oral administration SXT for 7 weeks.

## Conclusion

In the present study, a comprehensive metabolic analysis after oral administration with SXT in T2DM rats was firstly reported. According to our results, SXT exerted a significantly protective effect on hyperglycemia, IR, dyslipidemia and inflammation in diabetes rats. UPLC-Q-TOF-MS-based metabolomics profiling together with the analysis of NF-κB/PI-3K/Akt pathways was applied to reveal significant alterations in T2DM rats after SXT intervention. As shown in Figure 11, the results suggested that SXT might exert the anti-diabetes effect by attenuating inflammation to ameliorate insulin resistance and then improving the uptake and metabolism of glucose. Our study provided a further understanding on the mechanism of SXT on anti-diabetes.

## Author contributions

XW designed the study, performed experiments, analyzed the data, and wrote the manuscript. JT, YS, SX, ES, and ZZ helped with performed experiments and analyzed data. SJ helped with design and review of the manuscript. JD and DQ designed the study, interpreted the data, and reviewed the manuscript.

### Conflict of interest statement

The authors declare that the research was conducted in the absence of any commercial or financial relationships that could be construed as a potential conflict of interest.
