# Supplementary material for: Sanhuang Xiexin Tang Ameliorates Type 2 Diabetic Rats via Modulation of the Metabolic Profiles and NF-κB/PI-3K/Akt Signaling Pathways
Source: Front Pharmacol. 2018 Aug 28;9:955. doi: 10.3389/fphar.2018.00955 (PMC6121076; doi:10.3389/fphar.2018.00955)
Supplement: Supplementary file 1 [file Data_Sheet_1.docx]

Supplementary Material

**Sanhuang Xiexin Tang Ameliorates Type 2 Diabetic Rats via Modulation of the Metabolic Profiles and NF-κB/PI-3K/Akt Signaling Pathways**

*Corresponding author: Prof. Shu Jiang, [jiangshu2020@126.com](mailto:jiangshu2020@126.com) (S. Jiang), Tel. / fax: +86 25 85811516

*Correspondence to these authors: Prof. Jin-aoDuan, [dja@njutcm.edu.cn](mailto:dja@njutcm.edu.cn) (J.A. Duan), Tel. / fax: +86 25 85811516

**1 Supplementary Figures and Tables**

**1.1 Supplementary Figure**


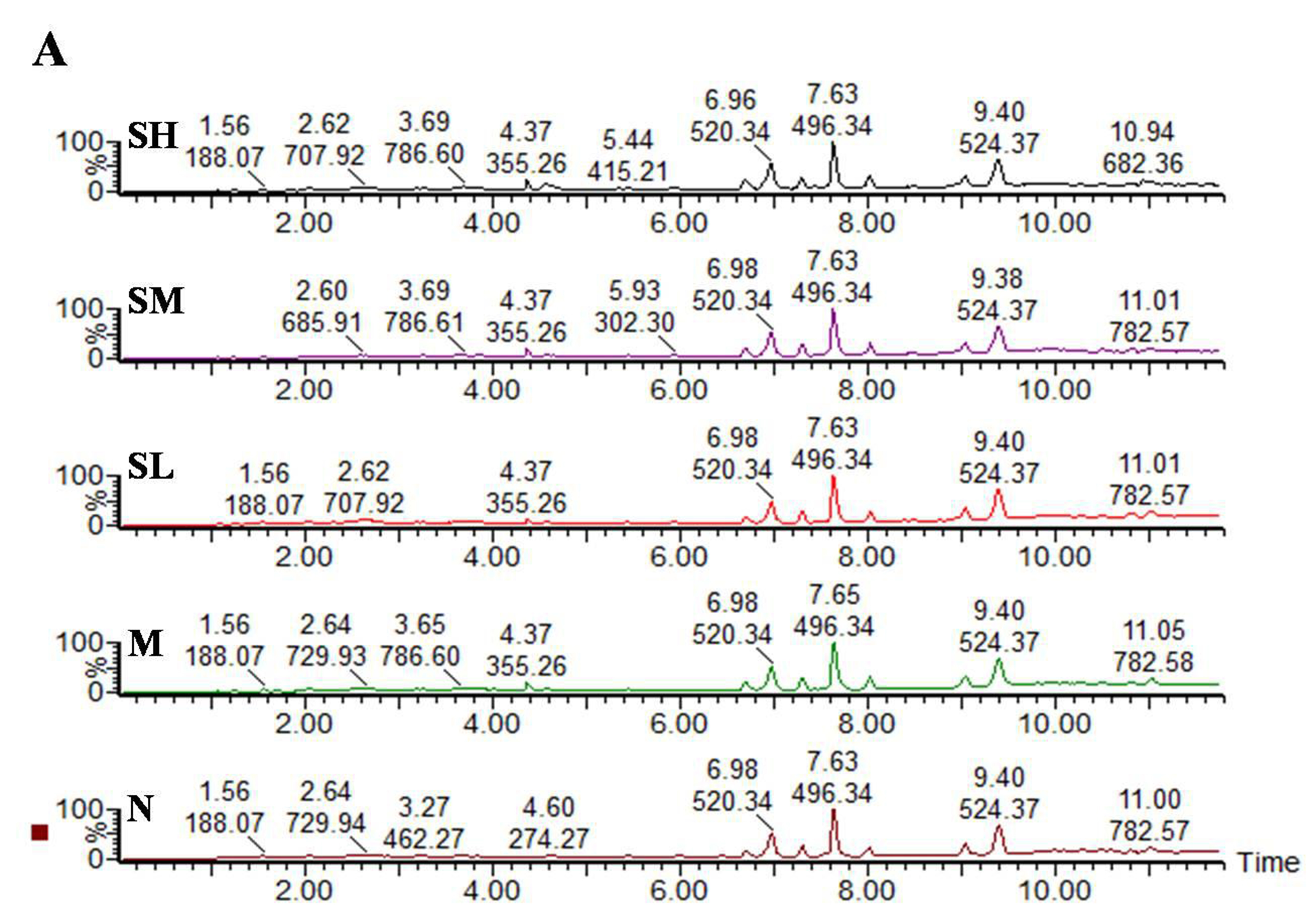

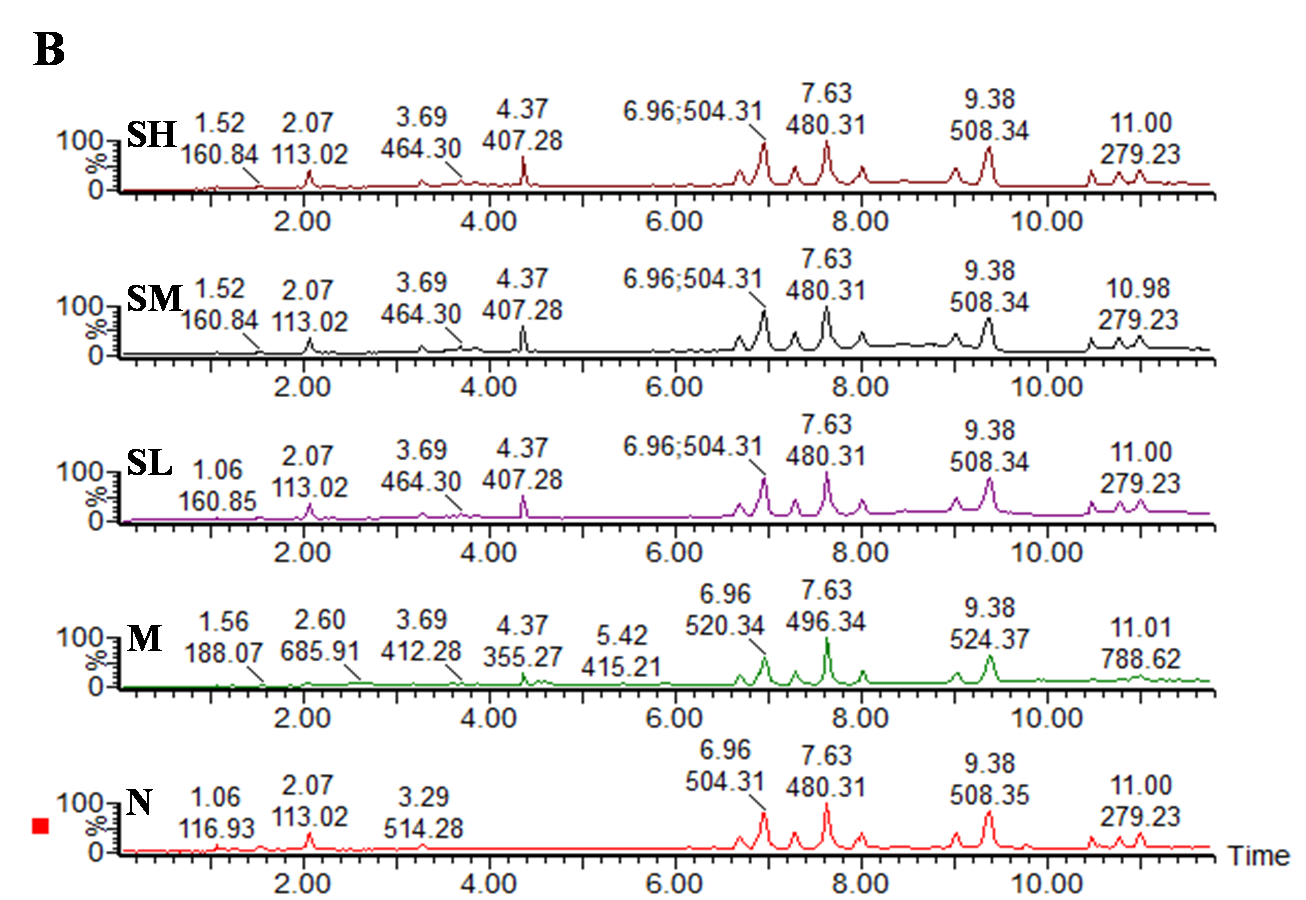

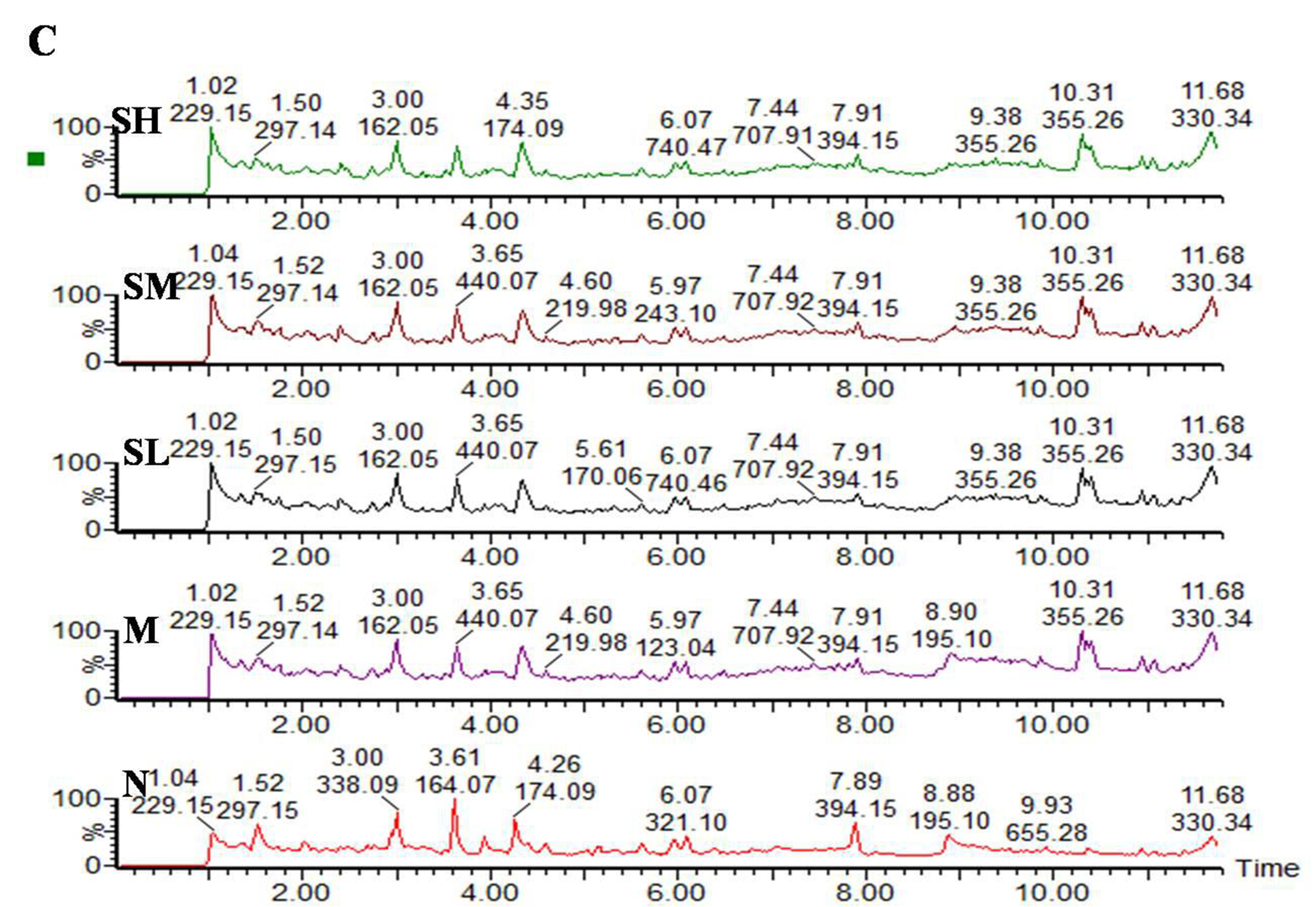

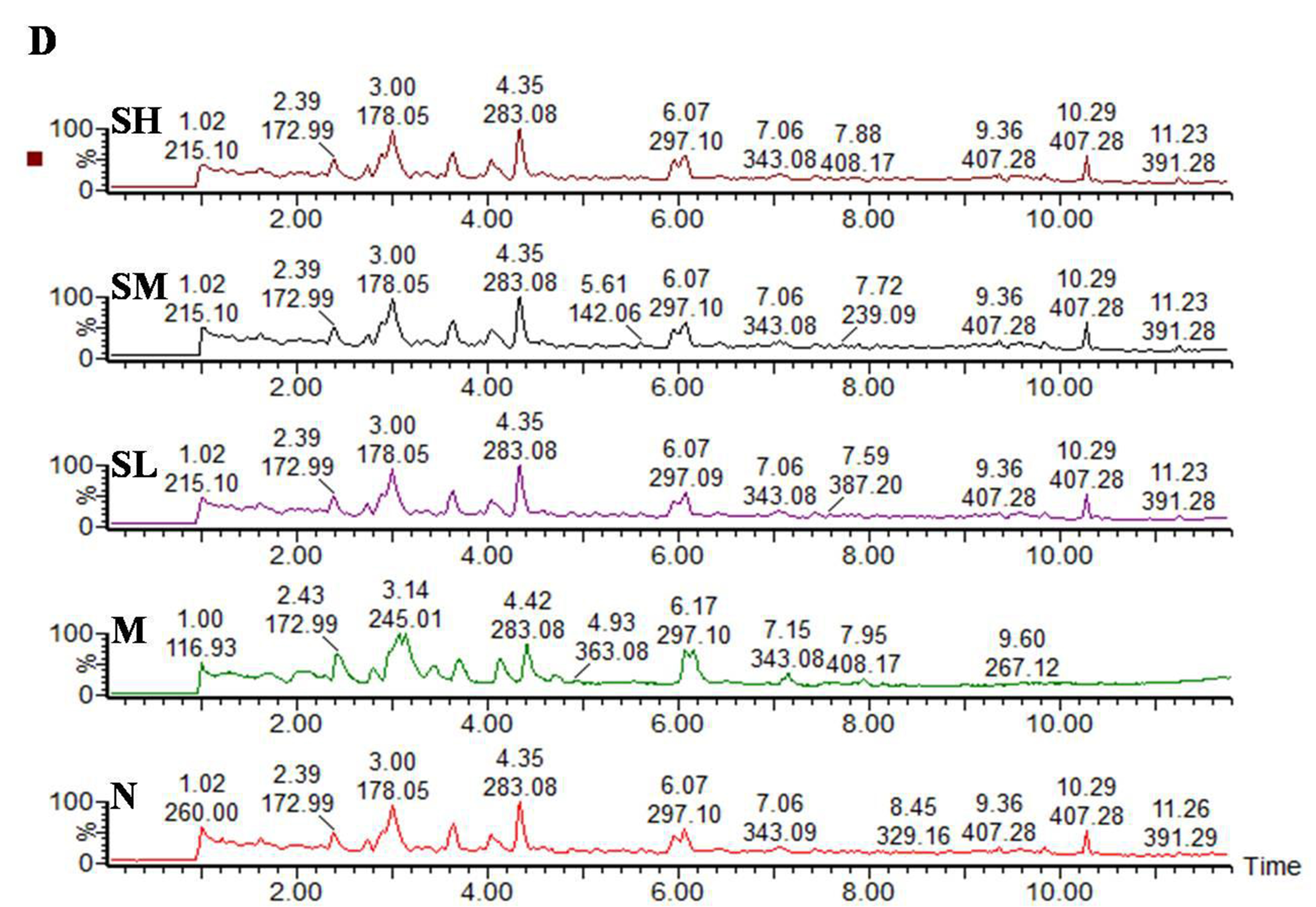


**Supplementary Figure 1. Metabolomic profiles by UPLC/Q-TOF-MS in positive and negative mode.** (A)Serum in ESI^+^ mode; (B) Serum in ESI^-^ mode; (C) Urine in ESI^+^ mode; (D) Urine in ESI^-^ mode. (Normal group (N), Model group (M), groups gavaged with metformin (Met), 5 g/kg SXT (SL), 10 g/kg SXT (SM) and 15 g/kg SXT (SH)

**1.2 Supplementary Table**

**Supplementary Table 1. Target genes and primer sequences used in this study**

| **Primer pairs** | **Nucleotide sequence (5’-3’)** | **Product length** | **Refs.** |
| --- | --- | --- | --- |
| **β-actin (S)** | GACCCAGATCATGTTTGAGAC | 238 | NM031144 |
| **β-actin (AS)** | GTAGCCACGCTCGGTCAG |  |  |
| **GAPDH (F)** | TGTGAACGGATTTGGCCGTA | 183 | NM017008.4 |
| **GAPDH (R)** | ACCAGCTTCCCATTCTCAGC |  |  |
| **PI-3K (F)** | GAGACACTGCTGATGGGACC | 180 | NM013005 |
| **PI-3K (R)** | AGTCTTTGCTGTACCGCTCC |  |  |
| **GLUT4 (F)** | GTTGGTCTCGGTGCTCTTAGT | 132 | NM012751 |
| **GLUT4 (R)** | CTCATGGATGGAACCCGCTC |  |  |
| **Akt (F)** | TGCAAAGGATGAAGTCGCC | 183 | NM017093 |
| **Akt (R)** | TCCTCCGTGAAGACTCGCT |  |  |
| **TNF-a (F)** | CGGTGCCTATGTCTCAGCCT | 233 | NM012675 |
| **TNF-a (R)** | TCCAGCTGCTCCTCCACTTG |  |  |
| **IL-6 (F)** | GAAATGTGGTCGGCAAGTCC | 198 | NM001008725 |
| **IL-6 (R)** | CTGGGATGCAGGGTGAGTTC |  |  |
| **CRP (F)** | CTTACGCTACCAAGACGAGC | 181 | NM017096 |
| **CRP (R)** | CCGTCAAGCCAAAGCTCTAC |  |  |
| **NF-κB (F)** | CAAGATCTGCCGAGTAAACC | 178 | NM199267 |
| **NF-κB (R)** | TCGGAACACAATGGCCACTT |  |  |
| **IL-1β (F)** | AGCCAACAAGTGGTATTCTCC | 196 | NM031512 |
| **IL-1β (R)** | TGAAGACAAACCGCTTTTCCA |  |  |

**2 Supplementary**

The detailed conditions during biomarker identification were as following: for both positive and negative ion modes, the condition was selected as follows: Initial retention time: 0.00; Final retention time: 12.00; Low mass: 100.00; High mass: 1000; XIC window (Da): 0.05; Peak Width at 5% Height (seconds): 1.00; Peak-to-Peak baseline noise: 0.00; Marker intensity threshold (counts): 10; Mass window: 0.05; Retention time window: 0.10; Noise elimination level: 20.
